# High-Level Extracellular Expression of a New β-N-Acetylglucosaminidase in *Escherichia coli* for Producing GlcNAc

**DOI:** 10.3389/fmicb.2021.648373

**Published:** 2021-03-11

**Authors:** Congna Li, Shun Jiang, Chao Du, Zhenghui Lu, Nisha He, Yuling Zhou, Sijing Jiang S, Guimin Zhang G

**Affiliations:** State Key Laboratory of Biocatalysis and Enzyme Engineering, College of Life Sciences, Hubei University, Wuhan, China

**Keywords:** β-N-acetylglucosaminidase, secretion, fermentation conditions optimization, enzymatic hydrolysis, GlcNAc

## Abstract

N-acetyl-β-D glucosamine (GlcNAc) is wildly used in cosmetics, nutraceuticals and pharmaceuticals. The traditional chemical process for GlcNAc production from chitin causes serious acidic pollution. Therefore, the enzymatic hydrolysis becomes a great promising and alternative strategy to produce GlcNAc. β-N-acetylglucosaminidase (NAGase) can hydrolyze chitin to produce GlcNAc. Here, a GH3 family NAGase encoding gene *BlNagZ* from *Bacillus licheniformis* was expressed extracellularly in *Escherichia coli* guided by signal peptide PelB. The recombinant BlNagZ presented the best activity at 60°C and pH 5.5 with a high specific activity of 13.05 U/mg. The BlNagZ activity in the fermentation supernatant can reach 13.62 U/mL after optimizing the culture conditions, which is 4.25 times higher than optimization before. Finally, combining BlNagZ with chitinase ChiA we identified before, chitin conversion efficiency to GlcNAc can reach 89.2% within 3.5 h. In all, this study provided not only a high active NAGase, and a secreted expression strategy to reduce the cost of production, which is conducive to the industrial application.

## Introduction

GlcNAc is the basic component of biological polysaccharides, such as murein, hyaluronic acid, glycoproteins, proteoglycans, glycosaminoglycans (GAGs), and other connective tissue building blocks of mammals. It can also inhibit bacteria and fungi’s growth, enhance human immunity, protect cartilage, bones and joints (Talent and Gracy, 1996). GlcNAc has been widely accepted as a health care product for delaying joint aging, cosmetic additives to improve wrinkles and pigmentation on the skin (Chen et al., 2010). As a drug, GlcNAc has also shown remarkable efficacy in the treatment of tumor, joint and intestinal inflammation (Shaoqing et al., 2014; Inokuma et al., 2016). Therefore, GlcNAc is in great demand in the market.

At present, GlcNAc is mainly prepared by chemical method, namely acid hydrolysis of chitin (Patil et al., 2013; García-Fraga et al., 2015), which cause serious environmental pollution. The enzymatic hydrolysis of chitin with mild reaction conditions, higher product purity and environmental friendliness becomes a great promising strategy for producing GlcNAc. The complete degradation of chitin to GlcNAc requires the synergistic action of chitinases and β-N-Acetylglucosaminidases (NAGases), that is, the colloidal chitin was hydrolyzed into chitin-oligosaccharides by chitinases, and further hydrolyzed into GlcNAc by NAGases (Du et al., 2019). Chitinase, such as ChiA, had achieved high-efficiency expression in our lab (Song et al., 2020). Therefore, it is essential to develop new NAGases with low cost for industrial use.

NAGases can be classified into four families of glycoside hydrolase (GH) 3, 20, 73 and 84, based on the character of amino acid sequence^1^. GH3 NAGases are usually distributed in several bacterial and metazoan cells, while members from the GH20 family are abundant in fungi and insects (Oliveira et al., 2018). Most GH20 NAGases are slightly alkaline (Zhou et al., 2017), which do not match the acid dissolution of colloidal chitin. NAGases of GH3 is mostly neutral and acidic, for example, the optimum pH of rNag3HWLB1 from *Sphingobacterium* sp. HWLB1 (Zhou et al., 2016) is 7.0, NahA from *Symbiobacterium thermophilum* (Ogawa et al., 2006) is 5.5, NagA from *Streptomyces thermoviolaceus* (Tsujibo et al., 1998) is 5.0 and NagA from *Trichinella spiralis* (Bruce and Gounaris, 2006) is 4.4, but their activity is generally low. It is well known enzymes’ cost is a major impediment to the industrial application (Tsujibo et al., 1998). Therefore, the discovery of weak acid NAGases with high activity is the prerequisite for enzymatic preparation of GlcNAc, and the secretion expression of high level would lower the enzyme cost.

Several NAGases were already expressed inside the cells of *E. coli*, which required cell lysis during the purification (Zhou et al., 2016; Du et al., 2019). For ease of the process, BsNagZ secretory expression in *Pichia pastoris* with multiple copy strategy has been conducted, which takes 14 days per fermentation (Jiang et al., 2019). Since the activity of BsNagZ is low, we need to find new NAGases with higher activity and better thermal stability. After searching for NCBI database based on the amino acid sequence of BsNagZ, we selected several homologous NAGases to study further. NAGase from *Bacillus licheniformis* (BlNagZ) was expressed. Although *P. pastoris* has the advantage of secretory expression, the fermentation period is too long (Jiang et al., 2019). Hence, we try to express BlNagZ in *E. coli* to shorten the fermentation period. The popular pET vectors, which facilitate the fusion of target proteins to the signal peptide PelB, are usually the first choice for enzyme secretory expression in *E. coli* (Samant et al., 2014). PelB with the property of periplasmic targeting has successfully guided the secretion of several proteins from *E. coli*. For example, Chitosanase (CSN) from *Aspergillus fumigatus* was successfully expressed in *E. coli* followed by extracellular secretion under the guidance of PelB (Huang et al., 2016). Cutinase from *Thermobififida fusca* was expressed in *E. coli* and excreted through the mediation of the PelB (Chen et al., 2008, 2011).

In this study, a NAGase gene *BlNagZ* from *B. licheniformis* was expressed in *E. coli* and secreted out of the cells via PelB signal peptide. The recombinant BlNagZ showed the maximum activity of 13.05 U/mg at pH 5.5 and 60°C. The expression level was improved by 4.25 times after optimizing the fermentation conditions. Combined with the reported chitinase (Song et al., 2020), recombinant BlNagZ can hydrolyze colloidal chitin to obtain GlcNAc with 89.2% conversion ratio within 3.5 h.

## Materials and Methods

### Plasmid, Strains, Chemicals and Medium

*B. licheniformis* WX-02 was offered by professor Chen Shouwen (Wei et al., 2010). The *E. coli* XL10-gold and BL21 (DE3) were purchased from Invitrogen (United States), and used for gene cloning and expression, respectively. The plasmid pET26b purchased from Stratagene was used as the expressive vector. The plasmids and strains were selected and maintained in LB medium containing 50 μg/mL kanamycin.

The DNA primers were synthesized by Sangon Biotech Co. (Shanghai, China). Restriction enzymes, Ex Taq DNA polymerase, T_4_ DNA ligase, and other related enzymes were obtained from TaKaRa (Dalian, China). The substrate 4-Nitrophenyl-N-acetyl-β-D-glucosamine (pNP-β-D-GlcNAc) was purchased from Sigma-Aldrich (St. Louis, MO, United States). All the chemicals and reagents were analytical grade or chromatographic pure grade.

### Construction of the Expression Plasmid pET26b-*BlNagZ*

A pair of primers, BlNagZ-F (5′ TTCGGATCCGGTGAAAAA CATTAGAAAAACCGTTATTTTTG 3′) and BlNagZ-R (5′ AGT GCGGCCGCCTGATAACTTGGAACTTCCAATGTGATATT 3′) were synthesized to amplify *BlNagZ* using the genomic DNA of *B. licheniformis* WX-02 as template. Then the PCR product was digested by restriction enzymes of *Bam*HI and *Xho*I and cloned into the vector pET26b (Supplementary Figure 1). The recombinant plasmid, named pET26b-*BlNagZ*, was verified by DNA sequencing (Sangon Biotech Co., Shanghai, China).

### Expression and Purification of Recombinant *BlNagZ*

The recombinant plasmid pET26b-*BlNagZ* was transformed into *E. coli* BL21 (DE3). The recombinant strain BL21 (DE3)-pET26b-*BlNagZ* was grown at 37°C overnight. Then the culture was transferred into 50 mL TB medium containing 50 μg/mL kanamycin, and grown at 37°C. When the *OD*_600_ of the culture reached 3.0, IPTG was added to a final concentration of 0.5 mM to induce protein expression. After 32 h, the fermentation supernatant was collected, and the target protein was detected by SDS-PAGE.

To purify the target protein, the fermentation supernatant was filtered by 0.45 μm filter membrane, and loaded into a 5 mL His-Trap column pre-equilibrated with buffer A (25 mM Tris-HCl, 150 mM sodium chloride, pH 7.5). Then washed by buffer B (buffer A with 10 mM imidazole), the target protein was eluted with buffer C (buffer A with 200 mM imidazole). Then eluent fractions containing the target protein were concentrated using Amicon Ultracentricon (Millipore) with a 30 kDa cut-off, and desalted via GE HiTrap desalting column to exclude imidazole. The purified BlNagZ were detected by SDS-PAGE. The concentration of protein was determined by the Bradford assay using bovine serum albumin (BSA) as the standard.

### Enzyme Assays

The activity of purified BlNagZ was determined by measuring the hydrolysis of pNP-β-D-GlcNAc (Yem and Wu, 1976), which was dissolved in 50 mM phosphate buffer (pH 5.5) solution at a concentration of 1 mM. In the reaction, 10 μL of protein fraction was mixed with 90 μL of 1 mM pNP-β-D-GlcNAc and incubated at 60°C for 10 min. The reaction was quenched with 200 μL of 200 mM sodium carbonate. The activity was determined in 96 well plates by measuring the absorption at 405 nm of p-Nitrophenol. One unit of activity is the amount of enzyme that released 1 μmol p-Nitrophenol per min at appropriate conditions.

### Effects of pH and Temperature on the Enzyme Activity and Stability

The optional pH of BlNagZ was determined in various 50 mM buffers over a pH range from 4.0 to 8.0. To analyze the pH stability, the enzyme samples were incubated in 50 mM buffers with different pH at 4°C for 12 h and the residual activity was assayed by the standard method.

The effects of temperature on the enzyme activity were determined at different temperatures ranging from 30 to 90°C in 50 mM sodium phosphate buffer (pH 5.5). The thermostability was tested by pre-incubating BlNagZ at 55, 60, and 65°C in 50 mM sodium phosphate buffer (pH 5.5) for various times, respectively, and measuring the residual activity.

### Effects of Metal Ions and Organic Reagents on Enzyme Activity

Different metal ions were added to the reaction mixture at a 5 mM final concentration to test their effects on the enzyme activity of BlNagZ. The effects of 5% organic reagents on the activity of BlNagZ were determined under optimal conditions (pH 5.5, 60°C). The reaction was performed at 60°C for 10 min. The activity of the enzyme without any additions was set as 100%.

### Optimization of Induction Expression and Secretion Conditions

There are many factors that affect the expression and secretion of BlNagZ. To increase the secretion of BlNagZ, the fermentation conditions were optimized. First, to investigate the effect of IPTG concentration on protein expression, IPTG with different concentration of 0.5, 0.75, 1.0, and 1.25 mM was used to induce at 37°C. Then, to investigate the effect of temperature on protein expression, the induction was performed at different temperatures of 18, 25, 30, and 37°C after the addition of 1 mM IPTG. Last, the effect of the different cell density on protein expression was researched, different initial induced cell density of *OD*_600_ 1.0, 1.5, 2.0, 2.5, 3.0, and 3.5 was performed to induced by 1 mM IPTG at 25°C.

Finally, all the factors including temperature (20, 25, 30, and 37°C), IPTG concentration (0.5, 0.75, 1.0, and 1.25 mM) and initial induced cell density of *O*D_600_ (2.0, 2.5, 3.0, and 3.5) were designed by software Orthogonal Design Assistant (Supplementary Table 1). The *OD*_600_ and enzyme activity of all the above samples were detected per 4 h after induction.

### Combinatory Hydrolysis of Chitin

To study the hydrolytic activity of BlNagZ, firstly, N, N′-Diacetylchitobiose was used as the substrate. The 500 μL reaction system (50 mM sodium phosphate buffer, pH 5.5), including 450 μL of 1 mM N, N′-Diacetylchitobiose and 50 μL enzyme (13.5 U/mL) was incubated at 55°C for 15 min. Then the mixture was filtered by 0.22 μm filter membrane after processed at 100°C for 10 min. The end products were analyzed by HPLC (Du et al., 2019).

Then the colloidal chitin was used as the substrate. The 500 μL reaction system (50 mM sodium phosphate buffer, pH 5.5), including 300 μL of 3% colloidal chitin and 200 μL enzyme (13.5 U/mL) was incubated at 55°C for 4 h. Then the products were analyzed by HPLC.

To increase the production of GlcNAc, the chitinase ChiA was used to hydrolyze colloidal chitin with BlNagZ. In the reaction system (50 mM sodium phosphate buffer, pH 5.5), 200 μL ChiA (15 U/mL) was added into the 400 μL of 30% colloidal chitin, and the mixture was incubated at 50°C for 3 h. Then, different amounts of BlNagZ (0, 0.65, 1.3, 1.95, 2.6 U, respectively) were added into the mixture, the total reaction was put to a constant volume of 1 mL using the same buffer. The products were analyzed by HPLC after 55°C incubation for 20 min.

## Results

### Gene Cloning and Sequence Analysis

The amino acid sequence of BsNagZ (GenBank: CAB11942.1) was subjected to protein BLAST in the NCBI database to search for new NAGases. Several new NAGases that show 30–70% amino acid similarity with BsNagZ were selected for heterologous expression and characterization, including BlNagZ of *B. licheniformis* WX-02. BlNagZ (GenBank: CP012110.1) showed 68.06% sequence identity to BsNagZ, which consists of 619 amino acids with a signal peptide located at 1st–26th aa of N terminal^2^. The theoretical molecular mass and pI of BlNagZ were 67.92 kDa and 8.75, respectively.

**FIGURE 1:**
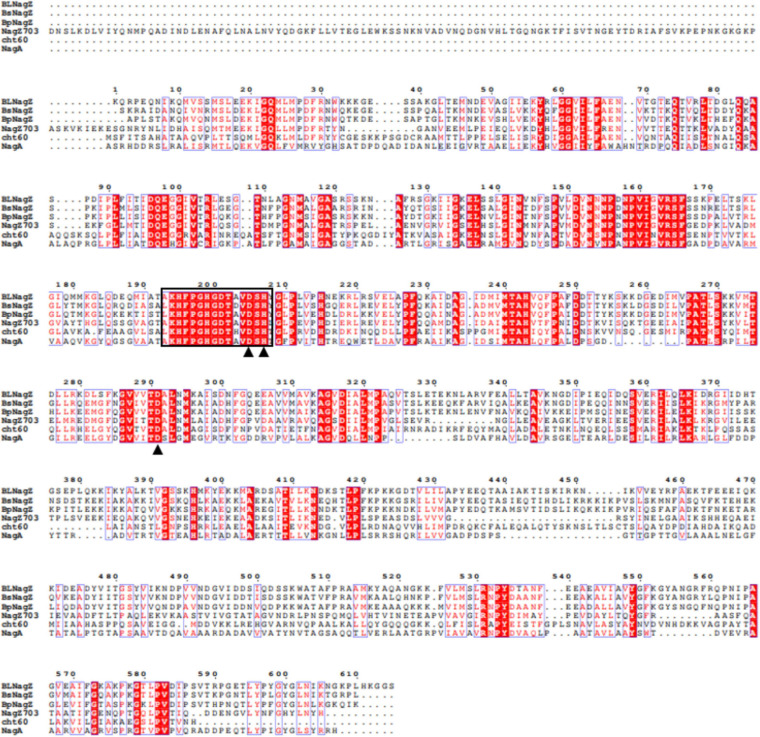
Multiple sequences alignments of BlNagZ with reported GH3 NAGases. Strictly conserved residues are indicated in white letters on a red background, and conservatively substituted residues are boxed. The catalytic active sites (D205, H207, D291) are indicated by solid triangle “▲”. The highly conserved sequence segment KH(F/I)PG(H/L)GX(4)D(S/T)H of GH3 family NAGase are Black box.

Through literature review, we found that 4 other NAGases with homology to BlNagZ have been studied in detail besides BsNagZ. These four NAGases are BpNagZ from *Bacillus pumilus* (GenBank: MK559425), NagZ703 from *Bacillus pseudofirmus* OF4 (GenBank: ADC51622.1), Cht60 from *Alteromonas sp.* strain O-7 (GenBank: D17399.1), and NagA from *Streptomyces thermoviolaceus* (GenBank: AB008771.1), which showed 67.26, 39.2, 33.81, and 31.2% sequence identity to BlNagZ, respectively. The amino acid sequence alignment between BlNagZ and the other NAGases was shown in Figure 1. The highly conserved sequence segment KH(F/I)PG(H/L)GX(4)D(S/T)H of GH3 family (Litzinger et al., 2010) was found in BlNagZ from K^194^ to H^207^, and the typical catalytic activity sites D205-H207-D291 was also found, which suggested BlNagZ was a N-acetylglucosaminidase of GH3 family.

### Expression and Purification of Recombinant BlNagZ

**FIGURE 2:**
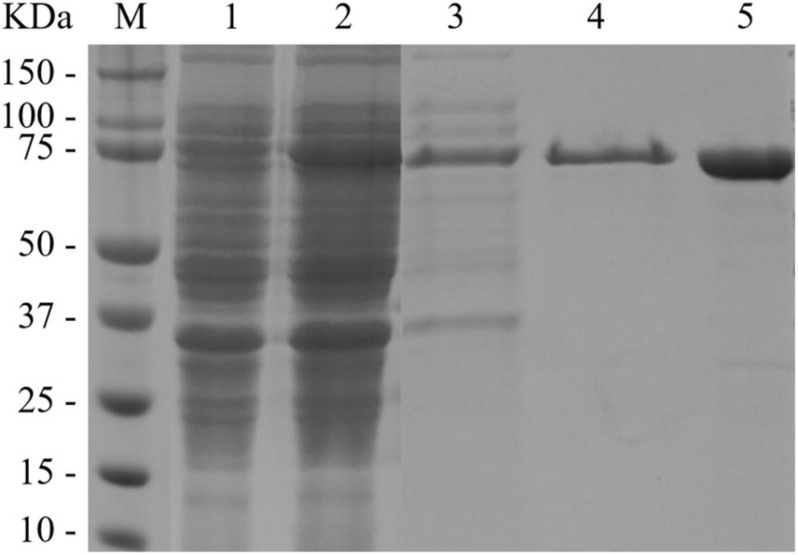
SDS-PAGE analysis of BlNagZ at each purification step. Lane M, prestained protein ladder; Lane 1, Bacterial precipitation of induced *E. coli* BL21 (DE3)-pET26b-BlNagZ cells; Lane 2, cell extract of induced *E. coli* BL21 (DE3)-pET26b-*BlNagZ* cells; Lane 3, fermentation supernatant of induced *E. coli* BL21 (DE3)-pET26b-*BlNagZ* cells; Lane 4, Purification on Ni-NTA affinity column; Lane 5, Purification on desalting column.

The recombinant BlNagZ was expressed soluble in *E. coli* BL21 (DE3) without obvious inclusion body. The SDS-PAGE analysis showed BlNagZ could secret into the medium and the purified BlNagZ migrated as a single band with a molecular mass of about 70 kDa, which was in accordance to the theoretically calculated mass (Figure 2). The pelB signal peptide was excised from BlNagZ when it was secreted. The specific activity of the enzyme after each purification step was calculated (Table 1). The results showed that the enzyme was stable during the steps of purification. The BlNagZ activity in the fermentation supernatant was 3.2 U/mL, and the specific activity reached 13.05 U/mg after purification.

**TABLE 1:** Purification steps of recombinant BlNagZ.

Purification	Total protein (mg)	Total activity (U)	Specific activity (U/mg)	Purification (-fold)
Crude enzyme	120	1073	8.95	1
Ni-column	20.09	244.69	12.18	1.36
Desalting column	7.03	91.81	13.05	1.46

### Biochemical Characterization of BlNagZ

**FIGURE 3:**
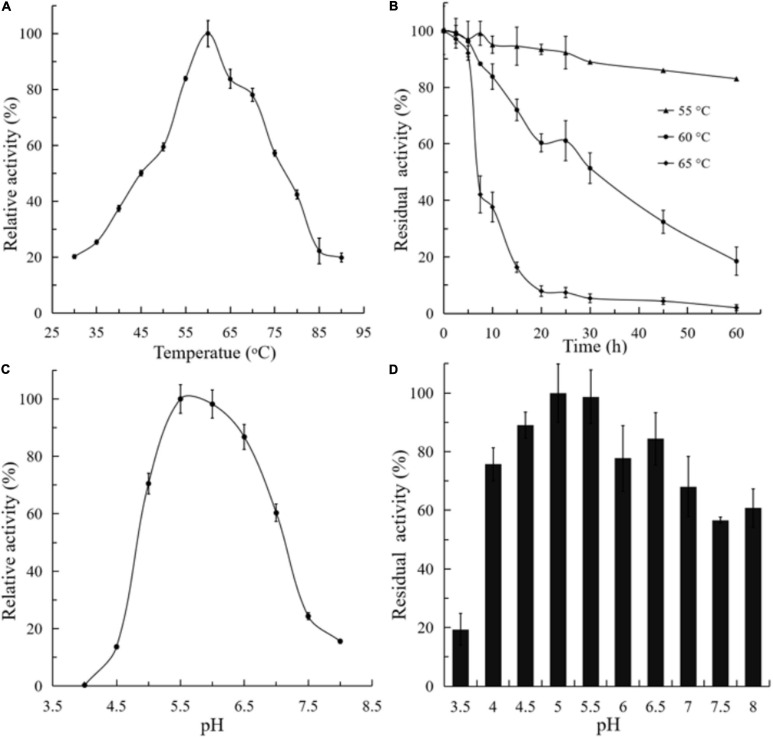
Effects of temperature and pH on recombinant BlNagZ activity. **(A)** Effects of temperature on the activity of BlNagZ. The reaction was conducted from 30 to 90°C in 50 mM phosphate buffer (pH 5.5); **(B)** effects of temperature on the stability of BlNagZ. The BlNagZ was pre-incubated at the indicated temperature in 50 mM phosphate buffer (pH 5.5) for 30 min. After pre-incubation, the residual activity of BlNagZ was measured at pH 5.5 and 60°C. **(C)** Effects of pH on the activity of BlNagZ. The buffers used were 50 mM citric phosphate (pH 4–8) at 60°C. **(D)** Effects of pH on the stability of BlNagZ. The recombinant enzyme was incubated at different pH for 12 h at 4°C. After incubation, the residual activity of BlNagZ was measured at pH 5.5 and 60°C. Each value of the assay was the arithmetic mean of triplicate measurements.

The activity and stability of purified BlNagZ were assayed at a temperature range from 30 to 90°C and pH range from 3.5 to 8. The optimum temperature is 60°C (Figure 3A). BlNagZ shows poor thermal stability at 60°C, only 50% of the residual activity remains after at 60°C for 30 min, and it keeps stable at 55°C with 90% residual activity after 30 min treatment (Figure 3B). The optimum pH of BlNagZ is 5.5, and it presents over 70% relative activity between pH 5 and 7 (Figure 3C). BlNagZ can retain over 70% residual activity between pH 4 and 6.5 after incubation at 4°C overnight (Figure 3D), which shows BlNagZ is stable in weak acidic conditions.

The effects of metal ions on the activity of BlNagZ toward pNP-β-GlcNAc were shown in Table 2. K^+^, Na^+^, Li^+^, Mg^2+^, and Mn^2+^ have no obvious effects on the activity of BlNagZ. But Cu^2+^ and Hg^2+^ almost abolish the enzymatic activity since Hg^2+^ and Cu^2+^ can act with free carboxyl groups in protein and form insoluble salts to inhibit the activity of most enzymes (Jorobekova et al., 2005). The addition of 5 mM EDTA didn’t affect the activity of BlNagZ, which suggested that BlNagZ isn’t a metal-dependent enzyme. Organic solvents such as methanol, alcohol and isopropanol showed slight inhibition, while formaldehyde inhibited most of the activity, because formaldehyde can denature enzyme by interacting with hydroxyl, sulfhydryl or amino groups of enzymes (Kamps et al., 2019). Glycerol and DSMO didn’t change the activity, which indicated that BlNagZ has better tolerance to organic solvents.

**TABLE 2:** Effects of metal ions and chemical reagents on BlNagZ activity.

Metal ions or chemicals	Concentration	Relative activity (%)	Metal ions or chemicals	Concentration	Relative activity (%)
No addition	0	100 ± 2.61	Hg^2+^ (HgCl_2_)	5 mM	0.026 ± 0.10
Li^+^ (LiCl)	5 mM	95.12 ± 2.08	EDTA	5 mM	88.43 ± 1.65
K^+^ (KCl)	5 mM	92.04 ± 8.54	Methanol	5%	90.76 ± 0.50
Na^+^ (NaCl)	5 mM	106.22 ± 7.27	Alcohol	5%	71.18 ± 0.18
Mg^2+^(MgCl_2_)	5 mM	94.28 ± 4.88	Isopropanol	5%	74.84 ± 0.19
Mn^2+^(MnCl_2_)	5 mM	96.45 ± 0.42	Glycerol	5%	100.4 ± 0.21
Ca^2+^ (CaCl_2_)	5 mM	78.85 ± 2.94	Formaldehyde	5%	7.47 ± 0.15
Cu^2+^ (CuCl_2_)	5 mM	0.51 ± 0.41	DSMO	5%	100.2 ± 0.32

*The assay was taken at 60°C and pH 5.5 with pNP-β-D-GlcNAc as substrate. No addition means the activity of BlNagZ was determined in buffers without the addition of any ions or chemicals. Each value of the assay was the arithmetic mean of triplicate measurements.*

### The Culture Condition Optimization to Improve the Secretion of BlNagZ

The recombinant strain BL21 (DE3)-pET26b-*BlNag*Z can express and secret BlNagZ into the medium, and the BlNagZ’s activity reached 3.2 U/mL when induced at 37°C with 0.5 mM IPTG for 32 h and initial *OD*_600_ of 2, respectively. In order to improve the extracellular yield of BlNagZ, we optimized the expression parameters, such as the concentration of IPTG, inducted temperature, and time, etc.

#### Effects of IPTG on the Secretion of BlNagZ

**FIGURE 4:**
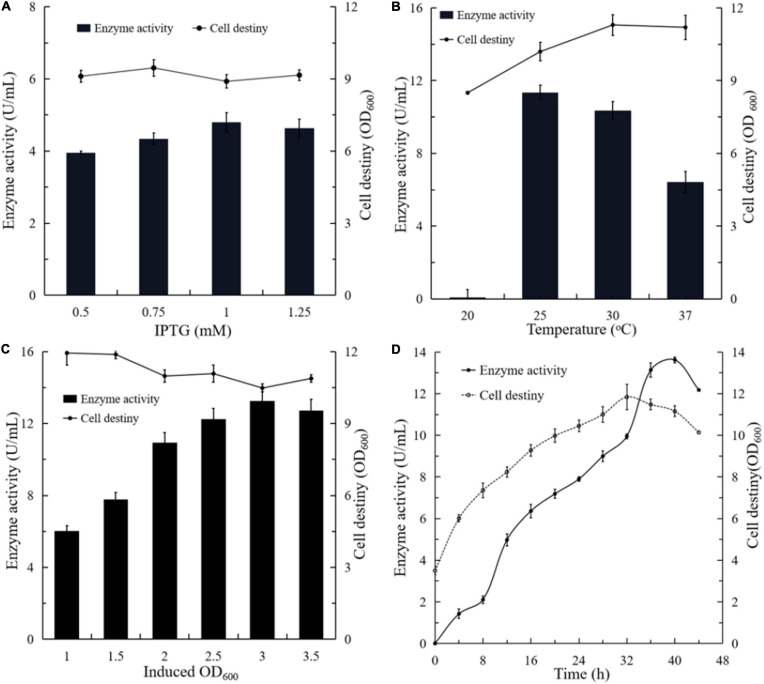
The effects of different culture conditions on the production of extracellular BlNagZ. **(A)** Concentraion of IPTG; **(B)** the induced temperature; **(C)** the initial induced *OD*_600_; **(D)** the optimal conditions determined by orthogonal experiment: 1 mM IPTG, 30°C and initial induced *OD*_600_ of 3.5.

By contrast, dose-dependent expression when using IPTG as an inducer is not possible since IPTG can enter the cell by active transport through the Lac permease or by permease independent pathways. Since the expression of Lac permease is heterogeneous and the number of active permeases in each cell is highly variable, protein expression does not respond predictably to IPTG concentration (Rosano and Ceccarelli, 2014). Different amounts of IPTG were added into the culture, respectively, when the *OD*_600_ reached 2.0 (Figure 4A). Compared to the control, the expression level of BlNagZ in the medium increased along with the increase of IPTG concentration. When the concentration of IPTG increased to 1.0 mM, the BlNagZ activity in the fermentation supernatant reached the highest of 5.3 U/mL at 36 h. Then its activity decreased when IPTG concentration increased to 1.25 mM. Meanwhile, different concentrations of IPTG has no obvious effect on cell growth. Therefore, 1.0 mM IPTG was more conducive to the secretion expression of BlNagZ.

#### Effects of Temperature on the Secretion of BlNagZ

Temperature is also an important factor for recombinant protein production in *E. coli* (Fang et al., 2011). In general, *E. coli* could slow down the synthesis rate of recombinant proteins when growing at a lower temperature, thereby increasing its solubility. However, it would lead to lower protein yield. To optimize the temperature for the production of BlNagZ, the strain was induced by 1 mM IPTG at 20, 25, 30, and 37°C, respectively. The results showed that the activity of BlNagZ in fermentation supernatant was highest of 11.2 U/mL when induced at 25°C for 40 h, which was 1.1 higher than induced at 30°C, and 1.7 higher than induced at 37°C (Figure 4B). In comparison, BlNagZ was hardly secreted outside the cells at 20°C. Induction at 25°C led to better coordination between the protein synthesis and cell growth. There was little difference in cell density while the strain was cultured under 25, 30, and 37°C, respectively. Therefore, 25°C was the best-induced temperature.

#### Effects of the Initial Induced Cell Density on the Secretion of BlNagZ

We then optimized the initial induced cell density at the condition of 1 mM IPTG and 25°C. The results showed that the activity of BlNagZ in the supernatant increased with the increase of starting cell density, and up to the highest 13 U/mL at 3 of *OD*_600_ after induction of 42 h (Figure 4C). Within the initial induced *OD*_600_ range of 2.5–3.5, the secretion expression levels of BlNagZ were similar. It is feasible for induction when the initial *OD*_600_ is at the range from 2.5 to 3.5.

#### Optimization of Induction Expression Conditions by Orthogonal Experiments

According to the results of orthogonal experiments, temperature is the most important factor affecting the expression and secretion of BlNagZ while the initial induced *OD*_600_ had the minimal effect (Supplementary Table 1). In all, 1 mM IPTG induction at 25°C for 40 h with initial *OD*_600_ 3.5 would be the best fermentation condition for secretion of BlNagZ and its activity can increase to 13.62 U/mL from 3.2 U/mL (Figure 4D).

### Hydrolytic Colloid Chitin to Produce GlcNAc

**FIGURE 5:**
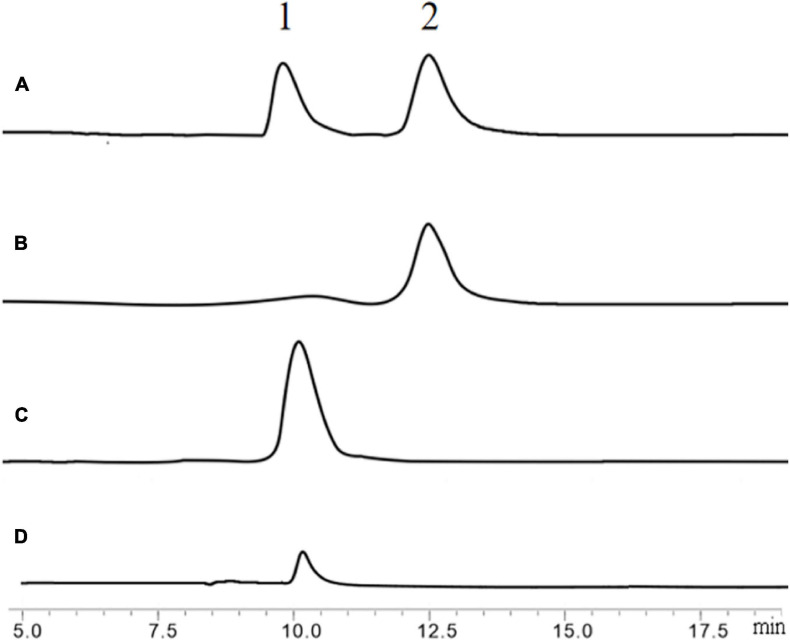
Hydrolysis of N, N′-Diacetylchitobiose by BlNagZ. **(A)** Standard of GlcNAc (peak 1) and N, N′-Diacetylchitobiose (peak 2); **(B)** the hydrolysis of N, N′-Diacetylchitobiose by BlNagZ at 0 min; **(C)** the hydrolysis of N, N′-Diacetylchitobiose by BlNagZ at 55°C for 15 min; **(D)** the hydrolysis of colloidal chitin by BlNagZ at 55°C for 4 h.

Firstly, N, N′-Diacetylchitobiose and colloidal chitin were used as substrates to verify the hydrolytic activity of BlNagZ. The results showed that BlNagZ could hydrolyze N, N′-Diacetylchitobiose into GlcNAc completely in 15 min (Figures 5A–C). However, the efficiency of this enzyme to directly hydrolyze colloidal chitin is very low, only a small amount of GlcNAc was produced (Figure 5D), which was consistent with previous reports (Jiang et al., 2019).

**FIGURE 6:**
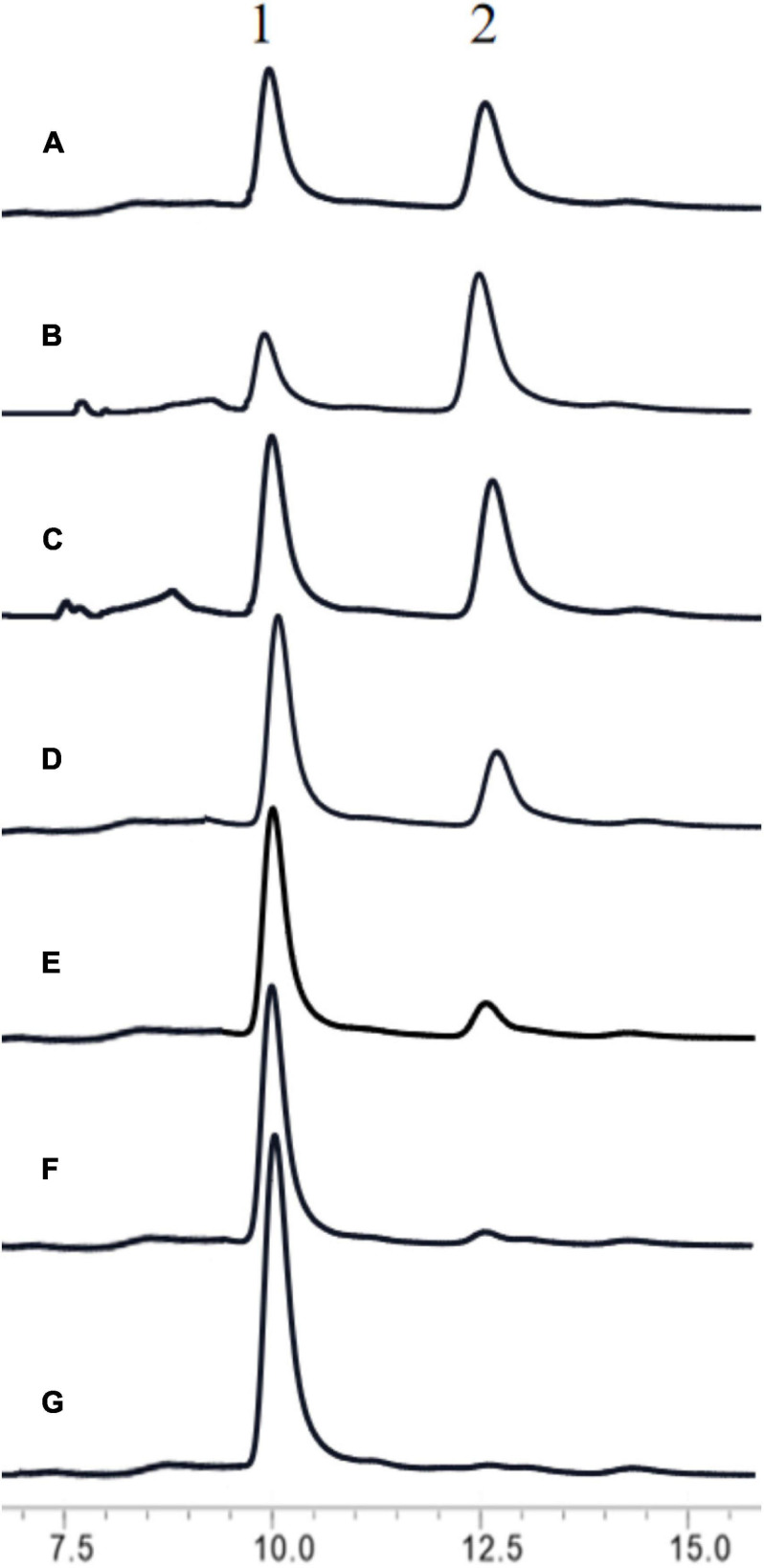
Hydrolysis of colloidal chitin by the combination of ChiA and BlNagZ. **(A)** Standard of GlcNAc (peak 1) and N, N′-Diacetylchitobiose (peak 2); **(B)** the hydrolysis product of colloidal chitin by ChiA at 50°C for 3 h; **(C–G)**: 0.0375, 0.65, 1.3, 1.95, 2.6 U of BlNagZ was added in 1 ml reaction system, respectively, for the second hydrolysis at 55°C for 30 min.

To increase the output of GlcNAc, 30% colloidal chitin was firstly hydrolyzed with chitinase ChiA (Song et al., 2020) for 3 h, and then hydrolyzed with different amount of BlNagZ for 30 min. The results showed that BlNagZ can effectively hydrolyze N, N′-Diacetylchitobiose produced by ChiA into GlcNAc, and 2.6 U/ml of BlNagZ could convert the hydrolysis product of ChiA into GlcNAc completely. Thus, the yield ratio of GlcNAc from 30% colloidal chitin could reach 89.2% within 3.5 h using the combination of ChiA and BlNagZ (Figure 6).

## Discussion

In this study, *BlNagZ* gene obtained from *B. licheniformis* WX-02 was expressed extracellularly in *E. coli* with PelB signal peptide. BlNagZ contained highly conserved sequence KH(F/I)PG(H/L)GX(4)D(S/T)H and the catalytic active center “D-H-D” that were typical in NAGases of GH 3 family. It can hydrolyze N, N′-Diacetylchitobiose and colloidal chitin to produce GlcNAc. These results demonstrated that BlNagZ was a NAGase of GH3 family.

Enzyme activity and stability, especially thermal and pH stability, must be considered in the industrial application. A few NAGases have good stability at 60°C or above, such as BsNagZ (Jiang et al., 2019), BpNagZ from *B. pumilus* (Du et al., 2019), CbsA from *Thermotoga neapolitana* (Kim et al., 2015), and NahA from *Symbiobacterium thermophilum* (Ogawa et al., 2006; Table 3). In this research, the optimum reaction temperature and pH of BlNagZ was 60°C and 5.5. And BlNagZ showed great thermostability, which remained 90 and 50% residual activity after pre-incubation at 55 and 60°C for 30 min, respectively. Similarly, BlNagZ had high activity over pH range from 5.5 to 6.5, and remained stable from pH 4.5 to 6.5. Acid-activity and acid-stability are desirable properties for enzymatic hydrolysis of chitin in an acidic environment. Therefore, BlNagZ is suitable to degrade chitin to GlcNAc in industry, and more importantly, its activity is the highest among the reported acid-stable NAGase.

**TABLE 3:** Biochemical characterization of the characterized GH3 NAGases.

Protein	Source	Optimal pH	Optimal temperature (°C)	Specific activity (U/mg)	*K*_*m*_ (μ mol/L)	*V*_*max*_ (μ mol/min)	Half-life (min, ^*o*^C)
BlNagZ	*B. licheniformis*	5.5	60	13.05	932	43.7	30, 60
BsNagZ	*B. subtilis* (Jiang et al., 2019)	6.0	60	2.9	1,130	39.27	30, 60
BpNagZ	*B. pumilus* (Du et al., 2019)	6.0	70	5.91	90.74	136.02	20, 70
CbsA	*Thermotoga neapolitana* (Kim et al., 2015)	8.0	75	–	–	0.3	–
NahA	*Symbiobacterium thermophilum* (Ogawa et al., 2006)	5.5	65	3.05	–	–	30, 60
NagA	*Streptomyces thermoviolaceus* (Tsujibo et al., 1998)	5.0	60	64.4	425.7	24.8	–
NagA	*Pseudomonas fluorescens* (Park et al., 2015)	8.0	37	5.3	130	245	60, 50
NagA	*Trichinella spiralis* (Bruce and Gounaris, 2006)	4.4	54	0.03	187	–	50, 54
rNag3HWLB1	*Sphingobacterium sp*. HWLB1 (Zhou et al., 2016)	7.0	40	–	1,120	19.88	15, 50

*“–” means the data haven’t been provided.*

Conventional expression of heterologous protein inside *E. coli* is complicated for industrial applications for cell disruption and protein purification. Here, BlNagZ was guided to the periplasmic space via PelB signal peptide with the property of periplasmic targeting, then secreted into the medium. According to Huang’s study, temperature, IPTG concentration, and induction time were determinant factors for the expression and secretion of CSN mediated by PelB (Huang et al., 2016). Therefore, we optimized the IPTG concentration, temperature, initial *OD*_600_ and induced time. BlNagZ activity of the supernatant increased to 13.62 U/mL, the expression of BlNagZ was almost 1.04 mg/mL, which was 4.25 times higher than optimization before (Figure 4) and about 70% of the BlNagZ can secret outside the cells. In other studies on the secretion expression of NAGases, only BsNagZ was secreted and expressed in *P. pastoris*, and the protein expression level reached 1.10 mg/mL at the 14th day (Jiang et al., 2019). Both of which have similar expression levels, but BlNagZ has higher activity and the fermentation time in *E. coli* is much shorter. There are also some proteins extracellular expressed in *E. coli* by PelB signal peptide. Such as *Candida antarctica* lipase B in the culture supernatant amounted to 0.55 mg/mL under the optimized conditions (Ujiie et al., 2016); The extracellular xylanase reached 1 mg/mL (Chang et al., 2017). Secretory expression greatly simplifies the process of protein purification. Moreover, the increase of the expression level reduces the production cost of the enzyme. Both are conducive to industrial application.

To our knowledge, NAGases are very inefficient at digesting chitin because of their cutting mode (Litzinger et al., 2010). And most of chitinases can not hydrolyze chitin into GlcNAc completely. Therefore, the high efficient conversion of chitin into GlcNAc requires the combinatory action of chitinase and NAGase (Nguyen-Thi and Doucet, 2016). For example, Fu et al. (2014) reported that NAGases from *Streptomyces coelicolor* combined with chitinase C could produce 27.8 mg/mL GlcNAc with a conversion rate of 92.6% after 24 h digestion. In Zhou et al.’s (2016) work, a small amount of GlcNAc was obtained by hydrolysis of chitin with Chitinase CtnSg, however, the yield of GlcNAc enhanced 3.74-fold by the synergy of CtnSg and GlcNAcase rNag3HWLB1. Wang et al. (2018) used BsChi from *B. subtilis* and OfHex1, a NAGase from the insect *Ostrinia furnacalis* to produce GlcNAc with 95% purity from pretreated crab shells with a yield rate of 60%. In our research, BlNagZ has the ability to hydrolyze chitin and produce GlcNAc with very low efficiency (Figure 5D). Combining with chitinase ChiA from *B. licheniformis* (Song et al., 2020), the yield rate of GlcNAc increased to 89.2%. This is slightly less than Fu et al.’s (2014) 92.6% and Wang et al.’s (2018) 95% conversion ratio. But the reaction time greatly reduced from more than 24 to 3.5 h by using enzymes with higher activities, which is also far lower than our previously reported 12 h (Song et al., 2020).

Usually, high substrate concentration would inhibit the enzyme activity. While in industry application, a high concentration of substrate can decrease the reaction volume, and then lower the production cost. It was found that ChiA can act on 30% (w/v) colloidal chitin with a high conversion ratio (Song et al., 2020). In this study, ChiA and BlNagZ are also combined to hydrolyze 30% colloidal chitin. Since chiA can efficiently hydrolyze chitin into (GlcNAc)_2_. Then BlNagZ can efficiently hydrolyze (GlcNAc)_2_ to GlcNAc. This greatly shortens the hydrolysis time by taking full advantage of these two enzymes. After hydrolysis by ChiA for 3 h, the substrate was then digested by BlNagZ for another 30 min, the conversion ratio of GlcNAc reached the maximum of 89.2%. Although the conversion rate was not the highest, the reaction time was greatly reduced. In addition, we also reduced the production cost of enzyme by improving the expression level of secretion, which laid a foundation for the preparation of GlcNAc by enzymatic hydrolysis of colloidal chitin in industry.

## Conclusion

This study is the first report to identify the *BlNagZ* gene from *B. licheniformis* WX-02 and expressed in *E. coli*. The secretory expression level of BlNagZ is the highest among all the reported cases. After optimization the expression conditions, the highest activity of BlNagZ in the fermentation supernatant reached 13.62 U/mL. Using high concentration colloidal chitin as substrate, the combination of recombinant BlNagZ and chitinase ChiA could produce GlcNAc with a yield of 89.2%. The whole hydrolysis process was controlled within 3.5 h, which is the fastest hydrolysis of colloidal chitin into GlcNAc speed at present.

## Data Availability Statement

The datasets presented in this study can be found in online repositories. The names of the repository/repositories and accession number(s) can be found below: https://www.ncbi.nlm.nih.gov/genbank/, CP012110.1.

## Author Contributions

SiJ and GZ designed the study. ShJ and CL completed the experiment and drafted the manuscript. CD and ZL provided technical support on experiment. YZ and NH helped to analyze the data. SiJ supervised the experiment process and revised the manuscript. GZ substantially revised the manuscript. All authors read and approved the final version of the manuscript.

## Conflict of Interest

The authors declare that the research was conducted in the absence of any commercial or financial relationships that could be construed as a potential conflict of interest.

## Footnotes

1 http://www.cazy.org/

2 http://www.cbs.dtu.dk/services/signalp

## Supplementary Material

Supplementary Figure 1 Construction flow chart of recombinant expression vector pET26B-*BlNagZ.*

Supplementary Figure 2 Dynamics analysis of recombinant enzyme BlNagZ.

Supplementary Figure 3 The analysis of BlNagZ secretion. Lane M, prestained protein ladder; Lane 1, fermentation supernatant without optimization; Lane 2, fermentation supernatant after optimization.

Supplementary Table 1 Results of orthogonal experiment.
