# Supplementary figures and images for: High-Level Extracellular Expression of a New β-N-Acetylglucosaminidase in Escherichia coli for Producing GlcNAc
Source: Front Microbiol. 2021 Mar 11;12:648373. doi: 10.3389/fmicb.2021.648373 (PMC7996098; doi:10.3389/fmicb.2021.648373)

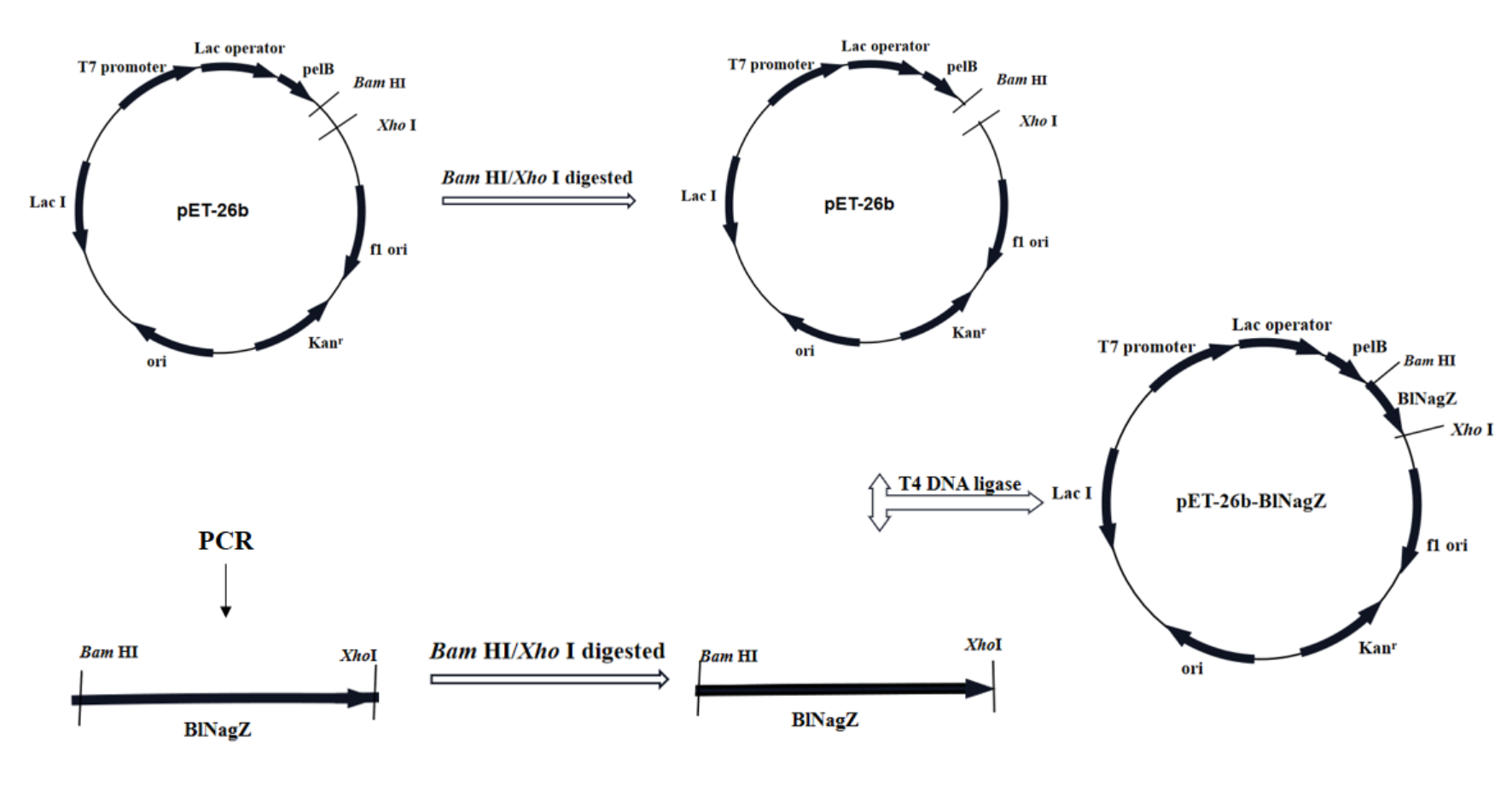

Supplement: Supplementary Figure 1 — Construction flow chart of recombinant expression vector pET26B-BlNagZ. [file Image_1.TIFF]

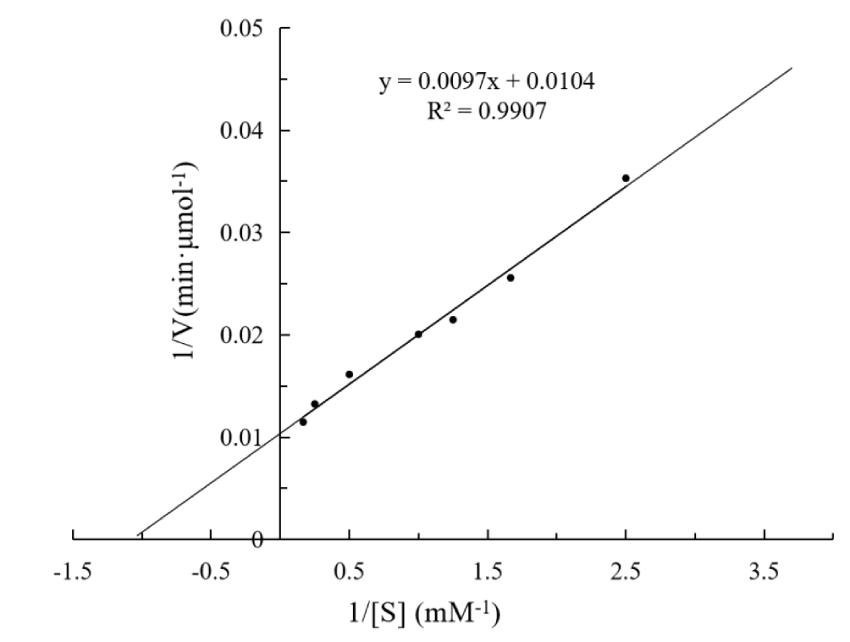

Supplement: Supplementary Figure 2 — Dynamics analysis of recombinant enzyme BlNagZ. [file Image_2.TIFF]

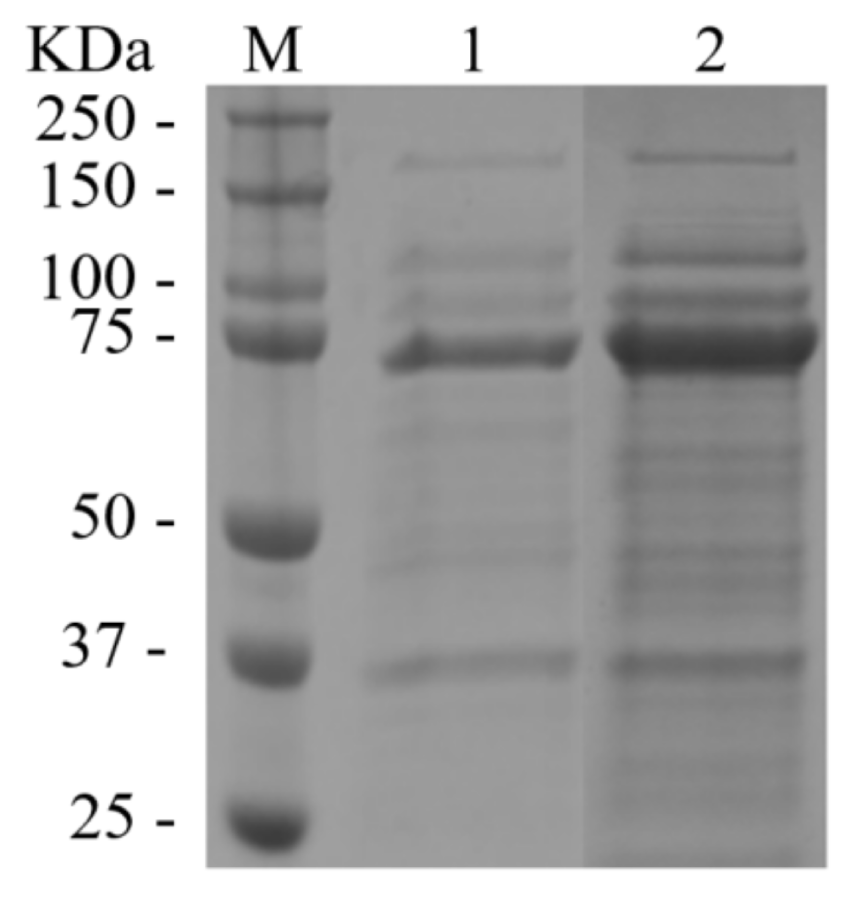

Supplement: Supplementary Figure 3 — The analysis of BlNagZ secretion. Lane M, prestained protein ladder; Lane 1, fermentation supernatant without optimization; Lane 2, fermentation supernatant after optimization. [file Image_3.TIFF]
