# Supplementary material for: High-Level Extracellular Expression of a New β-N-Acetylglucosaminidase in Escherichia coli for Producing GlcNAc
Source: Front Microbiol. 2021 Mar 11;12:648373. doi: 10.3389/fmicb.2021.648373 (PMC7996098; doi:10.3389/fmicb.2021.648373)
Supplement: Supplementary Table 1 — Results of orthogonal experiment. [file Table_1.DOCX]

**Table S1 Enzyme activity varies with time in orthogonal experiment**

| **factor** | **IPTG**  **(mM)** | **Temperature**  **(^o^C)** | **initial induced OD_600_** | **Enzyme activity (U/mL)** | | | | | | | |
| --- | --- | --- | --- | --- | --- | --- | --- | --- | --- | --- | --- |
|  |  |  |  | **16 h** | **20h** | **24 h** | **28 h** | **32 h** | **36 h** | **40 h** | **44 h** |
| Test 1 | 0.5 | 20 | 2.0 | 0.08±0.01 | 0.12±0.03 | 0.15±0.05 | 0.021±0.04 | 0.28±0.03 | 0.39±0.07 | 0.25±0.06 | 0.18±0.06 |
| Test 2 | 0.5 | 25 | 2.5 | 2.60±0.04 | 4.39±0.11 | 5.37±0.08 | 6.13±0.21 | 8.44±0.46 | 9.18±0.56 | 10.94±0.26 | 10.01±0.16 |
| Test 3 | 0.5 | 30 | 3.0 | 1.85±0.03 | 3.11±0.13 | 4.28±0.09 | 5.25±0.18 | 6.06±0.42 | 7.10±0.24 | 8.28±0.57 | 7.87±0.17 |
| Test 4 | 0.5 | 37 | 3.5 | 1.17±0.40 | 2.14±0.15 | 2.69±0.06 | 3.01±0.08 | 3.78±0.81 | 4.64±0.08 | 5.31±0.40 | 4.81±0.34 |
| Test 5 | 0.75 | 20 | 2.5 | 0.11±0.04 | 0.19±0.08 | 0.26±0.11 | 0.31±0.04 | 0.37±0.04 | 0.43±0.04 | 0.41±0.11 | 0.35±0.08 |
| Test 6 | 0.75 | 25 | 2.0 | 3.09±0.22 | 5.58±0.18 | 5.83±0.28 | 6.6±0.32 | 8.7±0.21 | 10.38±0.11 | 12.23±0.35 | 10.81±0.14 |
| Test 7 | 0.75 | 30 | 3.5 | 2.78±0.03 | 3.54±0.31 | 4.84±0.27 | 5.44±0.45 | 6.88±0.11 | 8.35±0.57 | 9.84±0.38 | 8.74±0.16 |
| Test 8 | 0.75 | 37 | 3.0 | 1.43±0.40 | 1.95±0.15 | 2.77±0.06 | 3.68±0.18 | 4.88±0.56 | 5.74±0.28 | 6.000±0.50 | 5.58±0.34 |
| Test 9 | 0.1 | 20 | 3.0 | 0.15±0.05 | 0.21±0.03 | 0.28±0.04 | 0.31±0.03 | 0.37±0.04 | 0.42±0.10 | 0.48±0.05 | 0.43±0.05 |
| Test 10 | 0.1 | 25 | 3.5 | 4.67±0.17 | 6.36±0.23 | 7.59±0.18 | 9.37±0.31 | 11.62±0.27 | 13.14±0.17 | 13.62±0.2 | 0.43±0.05 |
| Test 11 | 0.1 | 30 | 2.0 | 2.78±0.07 | 4.03±0.14 | 5.67±0.05 | 7.34±0.32 | 8.62±0.27 | 9.87±0.06 | 11.22±0.41 | 10.02±0.14 |
| Test 12 | 0.1 | 37 | 2.5 | 2.14±0.06 | 2.89±0.17 | 4.43±0.32 | 5.68±0.18 | 6.68±0.75 | 7.14±0.31 | 7.78±0.17 | 7.23±0.47 |
| Test 13 | 0.125 | 20 | 3.5 | 0.21±0.11 | 0.25±0.08 | 0.29±0.09 | 0.33±0.04 | 0.40±0.06 | 0.46±0.03 | 0.44±0.05 | 0.41±0.08 |
| Test 14 | 0.125 | 25 | 3.0 | 3.12±0.03 | 4.23±0.19 | 6.04±0.24 | 7.61±0.08 | 9.79±0.18 | 11.45±0.43 | 12.71±0.15 | 10.71±0.32 |
| Test 15 | 0.125 | 30 | 2.5 | 2.54±0.04 | 3.89.±0.03 | 5.07±0.23 | 7.32±0.38 | 8.76±0.50 | 9.45±0.12 | 10.67±0.03 | 9.78±0.43 |
| Test 16 | 0.125 | 37 | 2.0 | 1.21±0.03 | 1.78±0.21 | 2.02±0.36 | 2.89±0.20 | 3.78±0.43 | 4.05±0.12 | 4.34±0.48 | 4.14±0.18 |
